# Partial greater trochanter osteotomy for hip reduction in total hip arthroplasty for high dislocated hip: a preliminary report

**DOI:** 10.1186/1471-2474-15-293

**Published:** 2014-09-04

**Authors:** Rui Yu Liu, Chuan Yi Bai, Qi Chun Song, Xiao Qian Dang, Yao Jun Wu, Kun Zheng Wang

**Affiliations:** Department of Orthopaedic, Second Affiliated Hospital, College of Medicine, Xi’an Jiaotong University, No.157, Xiwu Road, Xi’an, 710004 Shaanxi P.R. China

**Keywords:** Partial greater trochanter osteotomy, Hip reduction, High dislocated hip, Total hip arthroplasty

## Abstract

**Background:**

Hip reduction in total hip arthroplasty for high dislocated hips is difficult. Various femur osteotomy procedures have been used for hip reduction, but these methods increase operative time and risk of nonunion. We investigated the efficacy of a novel partial greater trochanter osteotomy technique for hip reduction in total hip arthroplasty for patients with high hip dislocation.

**Methods:**

Twenty-one patients (23 hips) with high dislocated hip were treated with total hip arthroplasty that included partial greater trochanter osteotomy, i.e., the upper 2/3 greater trochanter was resected, and the gluteus medius muscle attachment was spared. The clinical outcome was evaluated by comparing the Harris hip scores and radiographic exam results, obtained before surgery and at follow-ups.

**Results:**

Follow-ups of 21 patients ranged from 13 to 56 months. The mean Harris hip score increased from preoperative 55.0 (36–69) to postoperative 86.1 (71–93; *P* = 0.00). The average preoperative leg length discrepancy in patients with unilateral high hip dislocation was 46 mm (28–65 mm); postoperatively leg length discrepancy was less than 1 cm in 11 patients, between 1 and 2 cm in 8 patients, and more than 2 cm in 2 patients. The average leg lengthening at the time of surgery was 36 mm (24–54 mm). Trendelenburg’s gait changed from positive to negative in 20 hips by the last follow-up. No nerve injury occurred postoperative.

**Conclusion:**

Partial greater trochanter osteotomy is an effective method to render hip reduction in total hip arthroplasty for patients with high dislocation of the hip.

**Electronic supplementary material:**

The online version of this article (doi:10.1186/1471-2474-15-293) contains supplementary material, which is available to authorized users.

## Background

Total hip arthroplasty (THA) has proved to be an effective treatment for osteoarthritis or limited mobility secondary to high dislocated hip. However, it is a difficult procedure for surgeons because of the associated anatomical abnormalities [1–3]. Hip reduction of the high dislocated hip is a considerable challenge because of the long-term soft-tissue contracture and high neurovascular injury risk after THA.

Many surgical options are available for reduction of femoral head dislocation, including trochanteric [4], subtrochanteric [5, 6], lesser trochanter osteotomy [7], and greater trochanter osteotomy of the femur [8]. However, these techniques carry a risk of nonunion and recurrent dislocation [9–11]. The reported overall rate of complications has ranged from 15% to 40% [5, 12, 13]. In addition, the extended operative time required for osteotomy increases the risk of infection. Rehabilitation may also be prolonged to assure healing of the trochanteric osteotomy. Iliofemoral distraction with external fixators before THA, followed by traction and delayed reduction [14, 15], has been described as an alternative to femoral shortening osteotomy. However, these techniques are uncomfortable for patients.

Herein we describe a novel hip reduction method using partial greater trochanter osteotomy during THA for high dislocated hip, and assess the effectiveness and safety of this procedure.

## Methods

The Ethics Committee of our hospital, the Second Hospital Affiliated to Xi’an Jiaotong University, approved the study protocol. All the study participants provided written informed consent for the study.

### Patients

Twenty-one patients (23 hips, 5 men and 16 women) with high dislocated hip received partial greater trochanter osteotomy during THA between May 2008 and March 2013. Based on the Crowe classification [16], all the high dislocated hips of the patients were in class Crowe- IV. Nineteen hips had developmental dysplasia; the other 4 high dislocated hips were due to childhood hip infections. The average age of the patients at the time of surgery was 44.6 years (range, 19–59 years). The indications for THA were severe hip pain or limited mobility. One experienced surgeon (Dr. Wang) performed all the surgeries.

### Evaluation of clinical outcome

An author who was not involved in the surgery conducted the clinical evaluations. Radiographic data were obtained for all the patients prior to surgery and at follow-up examinations. Routine radiographs included anteroposterior and lateral hip views. Preoperative anteroposterior radiographs of hip joint were taken to assess the severity of the dysplasia and anatomical variations of the acetabulum and proximal femur. Computed tomography scan was performed to evaluate the bone stock around the true acetabulum and the shape of the proximal femoral cavity. The stability of the acetabular components was assessed radiographically using the method introduced by DeLee and Charnley [17], and that of the femoral components by the method of Gruen et al. [18]. The clinical outcome was judged based on the Harris hip score [19]. In addition, Trendelenburg’s sign was recorded to measure abductor muscle strength, and leg length was compared before and after surgery by measuring the distance from the anterior superior iliac spine to the medial malleolus.

### Surgical technique

**Figure 1:**
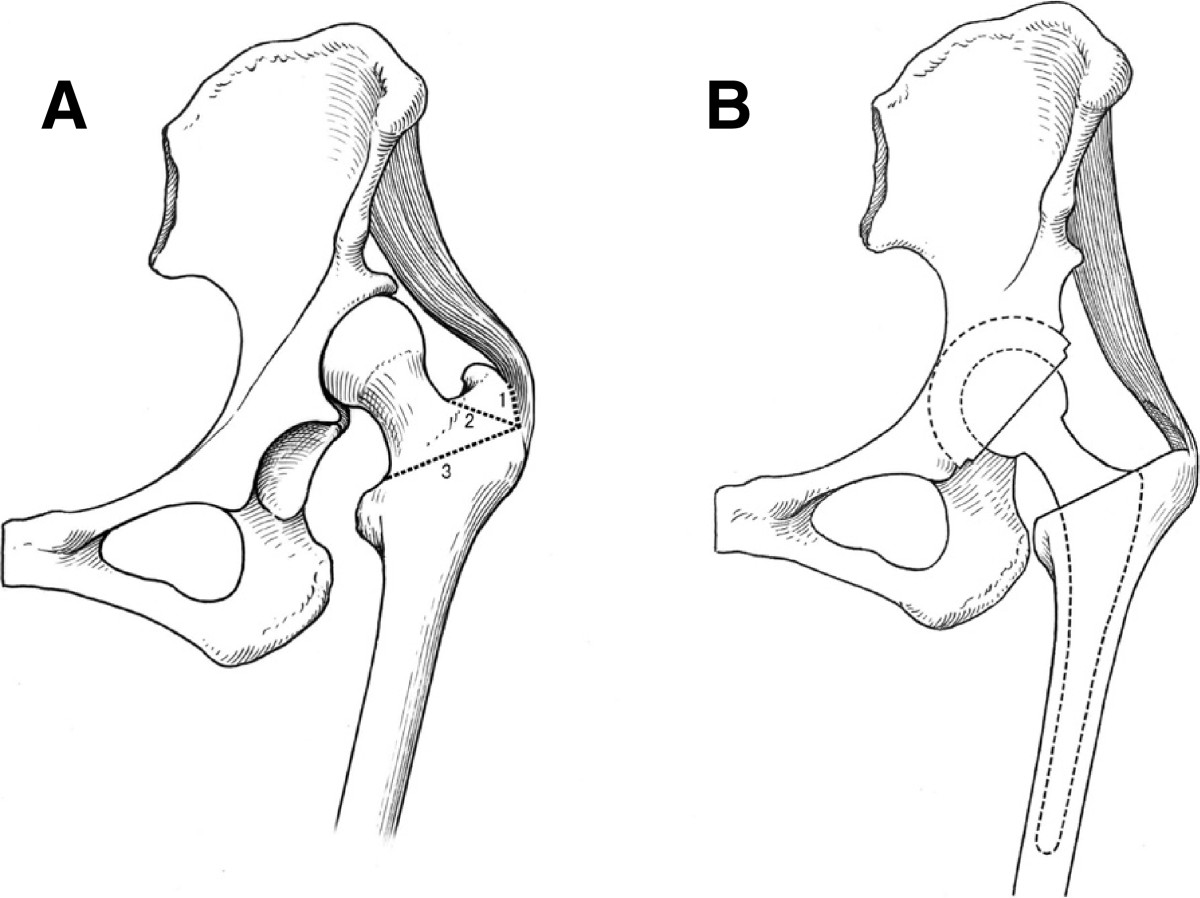
**Illustration of partial greater trochanter osteotomy. (A)** Before surgery. **(B)** After surgery. Intraoperative steps: 1) peel off the gluteus medius muscle attachment in the upper 2/3 of the greater trochanter; 2) partial greater trochanter osteotomy; 3) femoral neck osteotomy line.

The THAs were performed with patients in a lateral decubitus position, via a posterolateral approach. The superficial fascia was opened, the gluteus maximus was separated to expose the short external rotation, piriformis, and gluteus medius muscles. The gluteus medius muscle was pulled laterally. The short external rotator muscles and piriformis tendon were divided at their insertion site on the trochanter and separated carefully from the posterior capsule. The short external rotators and piriformis tendon were cut out at the side clinging to the intertrochanteric ridge. (To stabilize the hip and reduce the rate of dislocation, after insertion of the final hip prosthesis component, the short external rotator muscles and the piriformis tendon were repaired the same like the previous studies [20, 21]). The posteromedial edge of the gluteus medius was then identified, and the gluteus medius muscle tendon which attaches in the upper two-thirds of the greater trochanter was detached from the lateral facet greater trochanter with a bone stripper, after detaching the medial rim of the gluteus medius muscle. The upper two-thirds of the greater trochanter was resected in a posterior-to-anterior direction, which also released the gluteus medius muscle. During the procedure, care was taken to protect the attachment of the gluteus medius muscle tendon and minimize injury to it, and the attachment of the gluteus medius in the spared greater trochanter was purposely kept intact. (A diagram of the partial greater trochanter osteotomy is shown in Figure 1).

After the upper 2/3 greater trochanter resection, A trial reduction of the hip was performed to assess the tension of the soft tissue around hip joint. With the center of hip rotation displaced inferior-interiorly, the path of the gluteus medius muscle was changed from horizontal to oblique. The distance between its origin (the outer surface of the ilium) and insertion (the greater trochanter of the femur) was relatively shortened and the length of the gluteus medius muscle was relative lengthened, and this rendered the hip reduction easier.

The femoral neck osteotomy was conducted above the lesser trochanter. The proximal femoral canal was prepared, and the anteversion angle of the femoral neck was estimated. After this, the true acetabulum was identified, by dissection of the inferior part of the elongated capsule, and exposed. The joint capsule, scar fibrous tissue, and osteophytes around and within the acetabulum were carefully and completely removed. The acetabulum was reamed at a designated angle of abduction and antevertion, without perforating the inner medial cortex of the acetabulum. The orientation of the reconstructed acetabulum was considered in combination with the anteversion angle of the femoral prosthesis [22]. After the acetabular component was seated in the acetabulum, the femoral prosthesis was implanted. If the bone coverage of the acetabular component was less than 70%, a structural autograft was required from the resected femoral head, with bone screws for fixation to the upper border of the acetabulum.

Before performing the hip reduction, the hip adductor tension was determined by abducing the hip joint. Subcutaneous release of the adductor was done to reduce overly high adductor tension, which can limit the hip reduction and is apt to result in postoperative hip dislocation. To avoid neurovascular injury around the hip resulted from over-tension, the hip and knee joint was kept at 30° flexion during the traction reduction.

All components were cementless. The Bicontact® Universal Hip System (Aesculap, B. Braun, Germany) was used in 8 patients, and PROFEMUR® Total Hip System and LINEAGE® Acetabular Cup system (Wright, USA) was used in 6 patients. CombiCup® System and LCU® Hip prosthesis stems (LINK, Germany) was used in 6 patients. An acetabular cup and femoral prosthesis (models LB/TL and JA/W, respectively; Beijing Montagne Medical Device, China) was used in one patient.

### Postoperative rehabilitation

To protect the neurovascular tissue, flexion of the hip and knee at 30° was maintained with the patient on the bed during the first postoperative week. Low-molecular-weight heparin or rivaroxaban tablet was administered, and early functional exercise of the lower limb was encouraged to prevent venous thrombus. Partial weight-bearing was allowed one week postoperatively, except for patients who suffered intraoperative femoral fractures. The patients with intraoperative femoral fracture were allowed to walk after two months. Full weight-bearing was usually permitted 3 months after surgery.

### Statistical analyses

The Wilcoxon rank test was used to compare the Harris hip scores before and after surgery. SPSS software (Version 11, SPSS, Chicago, IL) was used for statistical analyses. A *P*-value < 0.05 was considered statistically significant.

## Results

**Figure 2:**
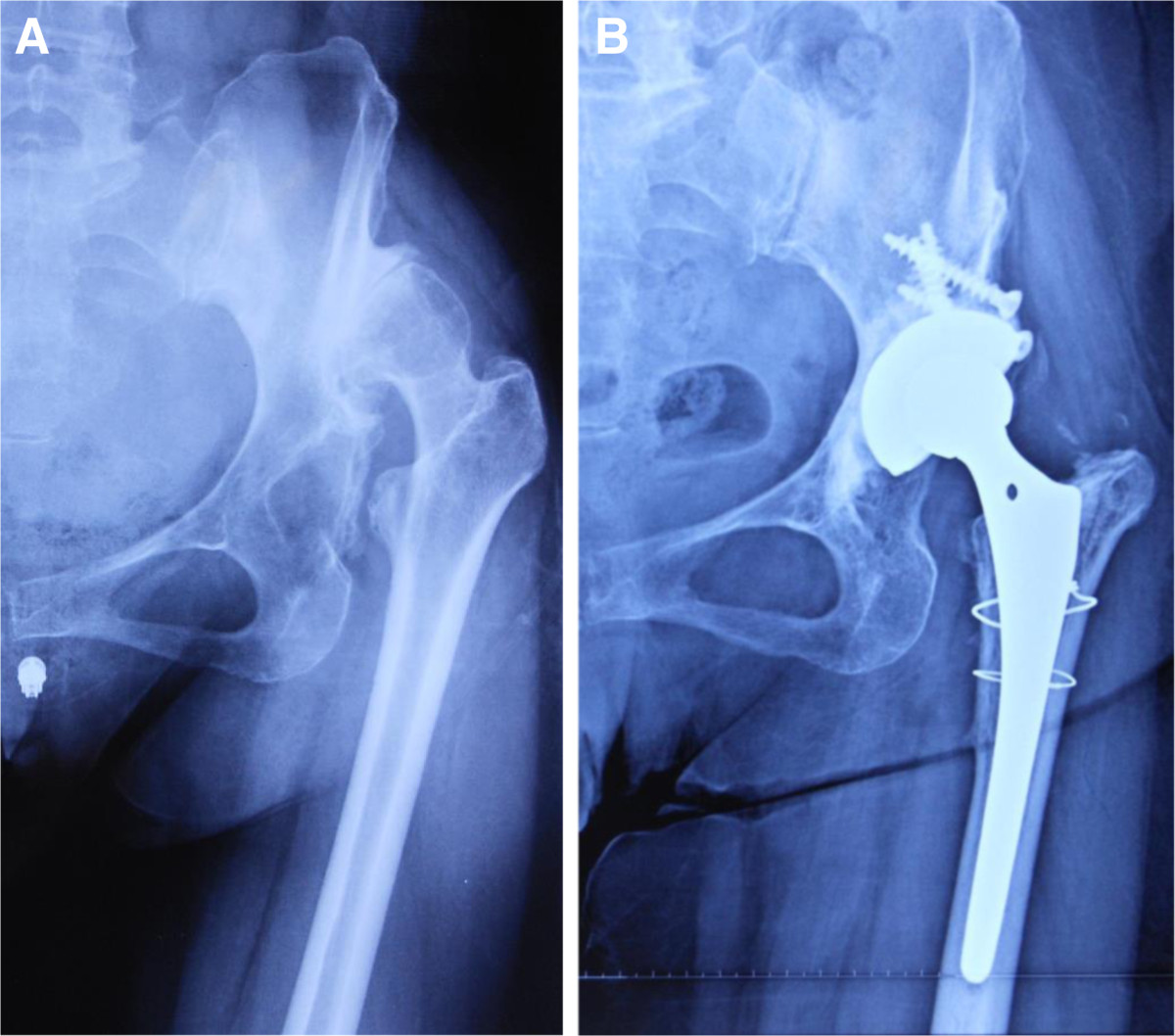
**Total hip arthroplasty performed in a 51-year-old woman with left hip high dislocation using partial greater trochanter osteotomy. (A)** Preoperative. **(B)** Four years after THA.

**Figure 3:**
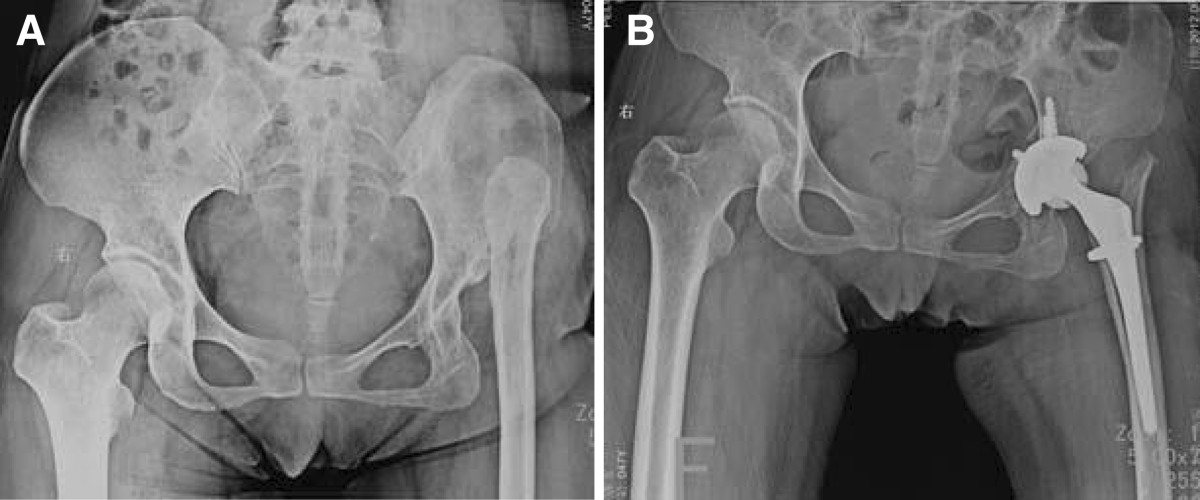
**Total hip arthroplasty performed in a 48-year-old woman with left high pathological hip dislocation using partial greater trochanter osteotomy. (A)** Preoperative. **(B)** Immediately postoperative.

The average follow-up period was 29 months (range, 13–56 months). The mean hip Harris score significantly improved from preoperative 55.0 (range, 36–69) to postoperative 86.1 (range, 71–93; *P* = 0.00). Preoperatively, the average leg length discrepancy in patients with unilateral high dislocated hip was 46 mm (range, 28–65 mm). Postoperatively, the leg length discrepancy was less than 1 cm in 11 patients, between 1 and 2 cm in 8 patients, and more than 2 cm in 2 patients. The average leg lengthening at the time of surgery was 36 mm (range, 24–54 mm). All the patients had a positive Trendelenburg’s sign before surgery; this persisted in 3 hips at the last follow-up examination.The radiographic evaluation showed no aseptic loosening of the components (Figures 2 and 3). There were 4 intraoperative fractures of the proximal femur, which fixed by cerclage wire intraoperative and healed 3 months after surgery. There was no dislocation, nerve palsy, infections, or deep venous thrombus in any of the patients.

## Discussion

Hip reconstruction in anatomical position can achieve optimal biomechanical results during THA in patients with high dislocated hip. However, for patients with high hip dislocation, reduction is implemented with great difficulty due to soft tissue contracture around the hip and the high risk of neurovascular injury. With the femur displaced proximally, the path of the gluteus medius muscle is changed from oblique to near horizontal and its length becomes shorter in the high dislocated hip [3]. Our osteotomy technique resected the upper two-thirds of the greater trochanter, and retained the insertion of the gluteus medius muscle in the spared greater trochanter. This surgical procedure releases the gluteus medius muscle and lengthens it, and changes the path of the gluteus medius muscle with hip reduction, reducing the distance between its origin and insertion and making the hip reduction easier. Concomitant with femoral head dislocation in patients with high dislocated hip, the adductor muscle is also contracted, which results in excessive adductor tension. For the sake of maintaining abductor-adductor balance and hip stability, subcutaneous adductor tenotomy was done to make hip reduction easier and avoid postoperative hip dislocation arised from excessive adductor tension.

Hip reduction without femur shortening osteotomy during THA in the high dislocated hip has been discussed by Zhao et al., Wu et al. and Yan et al. [23–25]. For some time we performed THA [26] in the high dislocated hip using extensive soft tissue release without femur osteotomy, as described by Wu et al. [24]. We found that this technique for hip reduction was very difficult and the risk of nerve injury was high. Furthermore, extensive soft tissue release reduces muscle strength and may result in hip instability [26]. Therefore, for cases of high hip dislocation, we developed a partial greater trochanter osteotomy method combined with less soft tissue release during THA.

Unlike the slide trochanter osteotomy [27] and the greater trochanter osteotomy [8], we do not perform a complete greater trochanter osteotomy, but just strip the attachment of the partial gluteus medius tendon in the upper two-thirds and resect this part of the greater trochanter. The insertion of the gluteus medius muscle in the left greater trochanter is spared. Thus, we do not need to repair the gluteus medius tendon or fix the greater trochanter. Anatomical study shows that there is no muscle or tendon attachment in some area of the lateral facet of the greater trochanter [28]. Therefore, our osteotomy technique causes less injury to the gluteus medius muscle tendon attachment. Even so, there is still some fear that this osteotomy may result in postoperative disattachment or disruption of the gluteus medius muscle insertion, or may cause pain of the greater trochanter. This was not observed in our short and mid-term follow-up in the present study.

Subtrochanteric femoral shortening osteotomy [4–6] is commonly used for neurovascular tissue protection and hip reduction during THA in patients with high dislocated hip. This technique carries a risk of nonunion, which will increase the risk of early loosening of the prosthesis, and the procedures are complex and require more operative time. More importantly, low-limb length is sacrificed, and disparity in low-limb length will aggravate limp, lumber scoliosis, and inclination of the pelvis [29]. The partial greater trochanter osteotomy method we describe herein needs no further fixation, is easy to perform, simplifies the surgical procedure, and saves operating time. Moreover, our technique avoids the risk of nonunion and malunion, and reduces rehabilitation time. Although the greater trochanter is partially resected, this has no influence on femoral prosthesis stability. However, lesser trochanteric osteotomy sacrifices a small part of the proximal femoral structure that is important for the rotational stability of the hip [7].

Hip abductor weakness is a very important issue for patients with high dislocated hip. With the hip reduction, normal hip biomechanics is restored and the abductor tension is strengthened, and further improvement of abductor tension depends on the abductor muscle. In the present series of patients, three hips showed a positive Trendelenburg’s sign in the one-year follow-up after THA. In these three hips, the abductor muscle was found to be weak intraoperatively.

Nerve palsy is a serious complication after THA in the high dislocated hip. No case of nerve palsy occurred in our series of patients, although there were 8 limbs that were lengthened more than 4 cm. The nerve injury after THA might not only be associated with the amount of limb lengthening [30], but also with the difficulty of the surgery [31]. Our method of partial greater trochanter osteotomy is relatively easy to perform and no extensive soft tissue release is done. The operation is especially easier than other various femur shortening osteotomies, and decreases the probability of nerve injury.

The proximal femoral morphology in high dislocated hip is not normal [32]; proximal femur deformity can cause the greater trochanter to partly cover the opening of the proximal femoral canal. Partial greater trochanter resection may contributive to expose the proximal femoral canal and femoral canal preparation. Intraoperative femoral fractures occurring in this study were related to mismatch between the proximal femoral canal and the selected prosthesis, it could be avoided by appropriate selection of a matched prosthesis or customized prosthesis.

The statistical power of our present study is limited by the small sample size and relatively short follow-up period although there is low incidence of high dislocated hip. Our results indicate that further studies in a larger series of patients and long-term follow-up are warranted to confirm the efficacy and safety of partial greater trochanter osteotomy during THA for patients with high hip dislocation.

## Conclusion

Partial greater trochanter osteotomy in THA is relatively easy to perform and effective in implementing hip reduction in patients with high dislocated hip. These early results show promise. To confirm the safety and efficacy of this technique, a study with a larger series of patients and long-term follow-up are warranted.

## Authors’ original submitted files for images

Below are the links to the authors’ original submitted files for images. Authors’ original file for figure 1 Authors’ original file for figure 2 Authors’ original file for figure 3

## Abbreviations

THA: Total hip arthroplasty.
