# Supplementary figures and images for: Partial greater trochanter osteotomy for hip reduction in total hip arthroplasty for high dislocated hip: a preliminary report
Source: BMC Musculoskelet Disord. 2014 Sep 4;15:293. doi: 10.1186/1471-2474-15-293 (PMC4174664; doi:10.1186/1471-2474-15-293)

**A**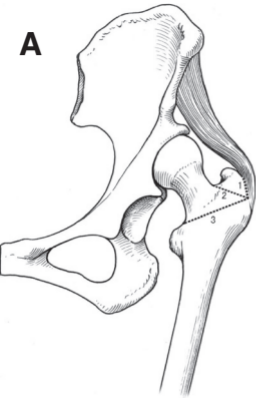**B**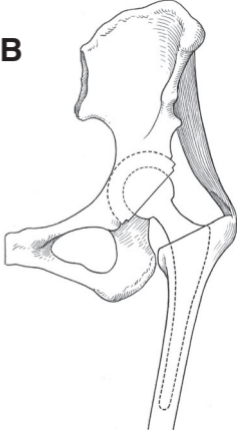

Supplement: Supplementary file 1 — Authors’ original file for figure 1 [file 12891_2014_2245_MOESM1_ESM.pdf]

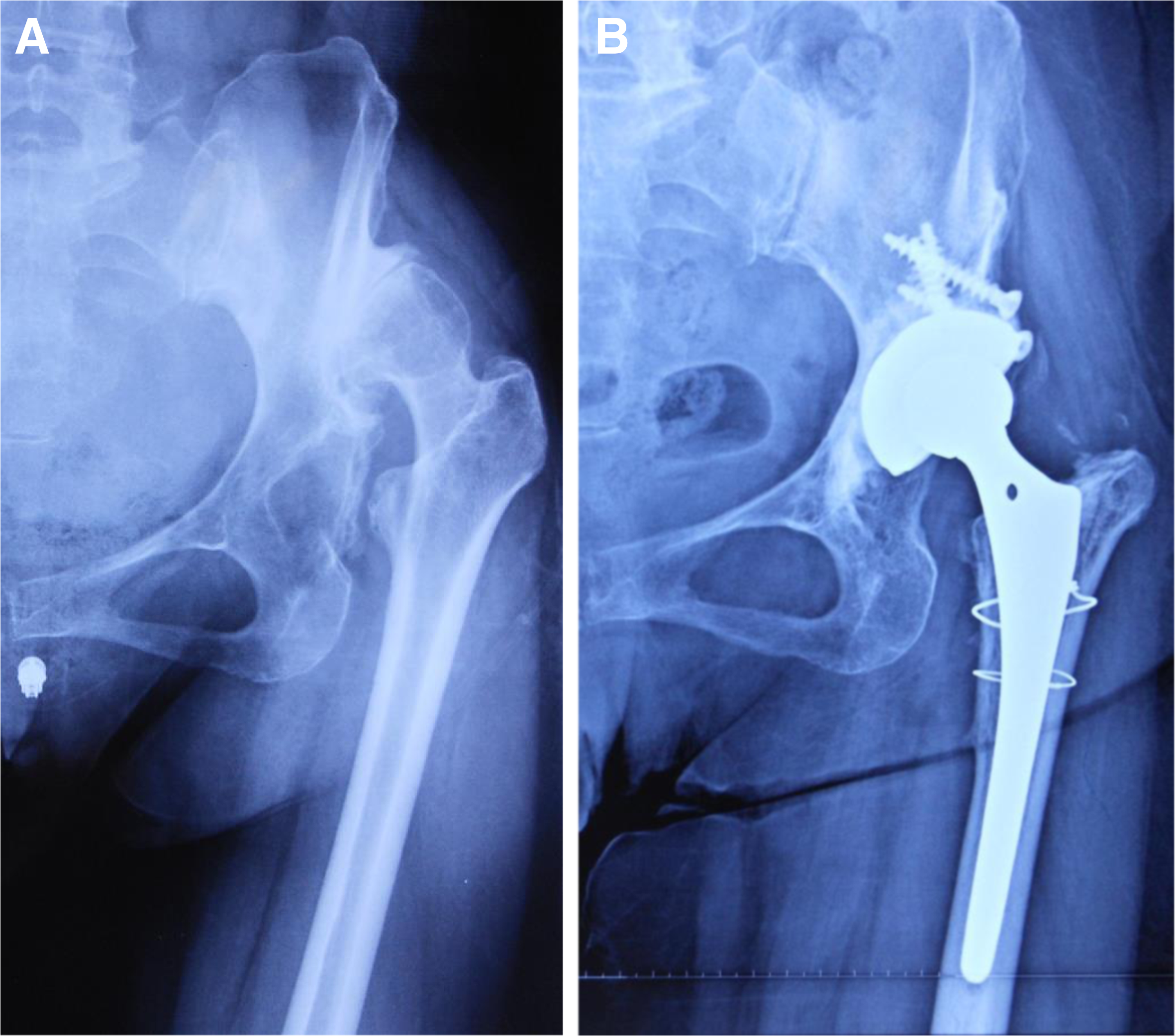

Supplement: Supplementary file 2 — Authors’ original file for figure 2 [file 12891_2014_2245_MOESM2_ESM.tif]

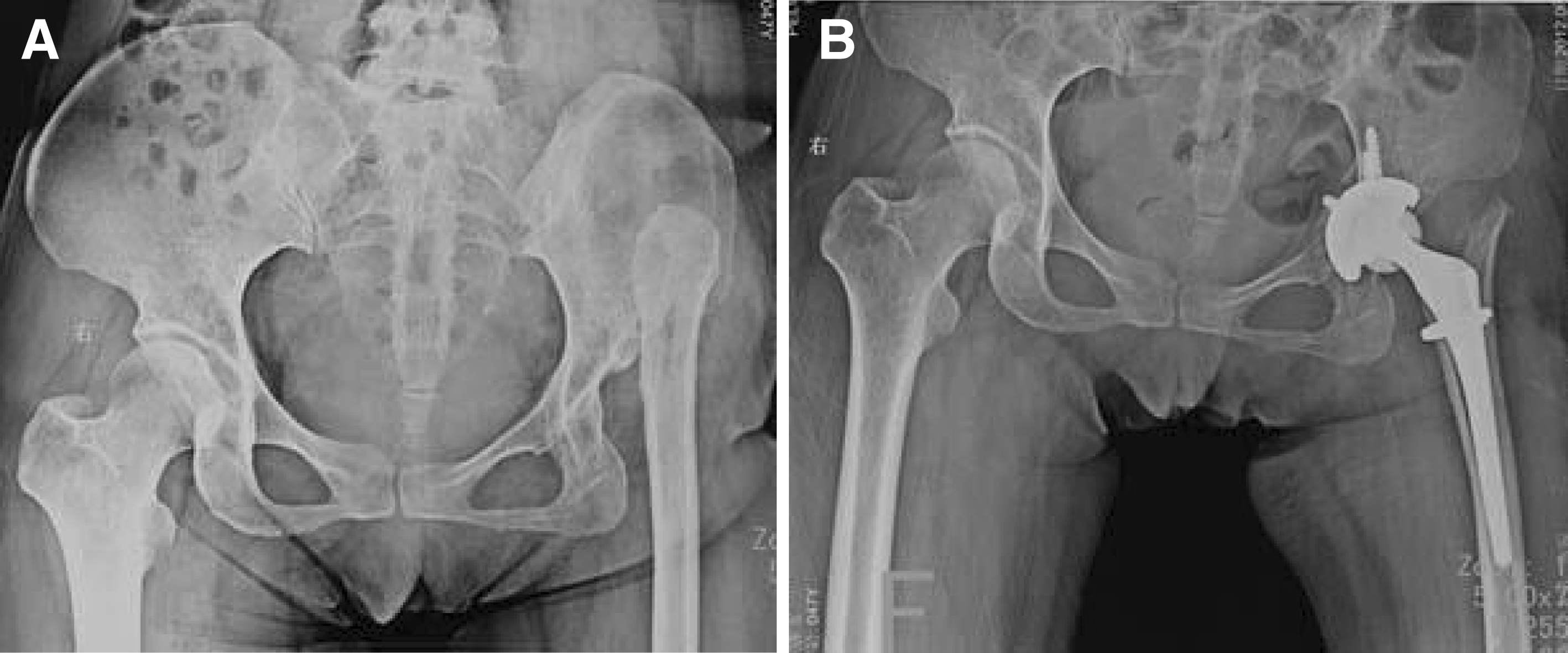

Supplement: Supplementary file 3 — Authors’ original file for figure 3 [file 12891_2014_2245_MOESM3_ESM.tif]
